# Single-Step Hydrolysis and Derivatization of Homocysteine Thiolactone Using Zone Fluidics: Simultaneous Analysis of Mixtures with Homocysteine Following Separation by Fluorosurfactant-Modified Gold Nanoparticles

**DOI:** 10.3390/molecules27072040

**Published:** 2022-03-22

**Authors:** Apostolia Tsiasioti, Constantinos K. Zacharis, Paraskevas D. Tzanavaras

**Affiliations:** 1 Laboratory of Analytical Chemistry, School of Chemistry, Faculty of Sciences, Aristotle University of Thessaloniki, 54124 Thessaloniki, Greece; atsiasioti@gmail.com; 2 Laboratory of Pharmaceutical Analysis, Department of Pharmaceutical Technology, School of Pharmacy, Aristotle University of Thessaloniki, 54124 Thessaloniki, Greece; czacharis@pharm.auth.gr

**Keywords:** homocysteine thiolactone, homocysteine, zone fluidics, *o*-phthalaldehyde, fluorosurfactant-modified gold nanoparticles

## Abstract

Herein, we report a new automated flow method based on zone fluidics for the simultaneous determination of homocysteine and homocysteine thiolactone using fluorimetric detection (*λ*_ext_ = 370 nm/*λ*_em_ = 480 nm). Homocysteine thiolactone is hydrolyzed on-line in alkaline medium (1 mol L^−1^ NaOH) to yield homocysteine, followed by reaction with *o*-phthalaldehyde in a single step. Derivatization is rapid without the need of elevated temperatures and stopped-flow steps, while specificity is achieved through a unique reaction mechanism in the absence of nucleophilic compounds. Mixtures of the analytes can be analyzed quantitatively after specific separation with fluorosurfactant-capped gold nanoparticles that are selectively aggregated by homocysteine, leaving the thiolactone analogue in solution. As low as 100 nmol L^−1^ of the analyte(s) can be quantified in aqueous solutions, while concentrations > 2 μmol L^−1^ can be analyzed in artificial and real urine matrix following 20-fold dilution. The percent recoveries ranged between 87 and 119%.

## 1. Introduction

Homocysteine thiolactone (HTL) is a well-known modifying factor of proteins, and its role in the pathogenesis of different diseases has started to be recognized [1,2,3,4]. HTL is a chemically reactive metabolite generated by methionyl-tRNA synthetase and cleared by the kidney [5]. There are numerous recent studies trying to elucidate the role of HTL in human health, including the oxidative status of liver and intestines [6], sperm function [7], blood vessel disfunction [8], and cardiovascular diseases [9].

HTL has, therefore, attracted the interest of analytical chemists and there are various methods in the literature reporting its determination in biological material, either alone [10,11,12,13,14,15,16] or in combination with HCY [17,18,19]. Due to the complexity of the biological matrices, the majority of the methods take advantage of the enhanced selectivity features of separation instrumental techniques, such as gas chromatography (GC) [11,13,17], liquid chromatography (HPLC) [15,16,18], and capillary electrophoresis [10,12,19,20]. Electrophoretic techniques offer low operational costs and high separation efficiency but generally low sensitivity. HTL/HCY can be detected directly using simple UV detection, but sensitivity enhancement to sub-micromolar levels requires preconcentration by either single-drop microextraction (SDME) [10,20], or by field-amplified sample stacking [10,12]. GC-MS is reported to be able to detect HTL/HCY selectively at micromolar levels with a derivatization/extraction step always being necessary to improve the volatility of the analytes [11,13,17]. HPLC is considered to be by far the most widely applied technique in bioanalysis and there are a couple of recent elegant reports on the analysis of HTL/HCY. For example, HTL/HCY were derivatized with *o*-phthalaldehyde on-column (the reagent was incorporated in the mobile phase), resulting in sharp peaks and fast elution. However, the stability of the reversed phase column under highly alkaline conditions (0.1 mol L^−1^ NaOH in the mobile phase) should always be of concern [13]. Alternatively, the analyte(s) can be determined by HPLC directly (UV at 240 nm) [16] or after post-column derivatization combined with cation exchange purification [15].

In our previous work, we have studied the selective reaction of HCY with *o*-phthalaldehyde (OPA) in highly alkaline medium under flow conditions using the concept of zone fluidics (ZF) [21]. Herein, we expand our work on investigating the potential of simultaneous determining of HCY and HTL based on the rapid alkaline hydrolysis of the latter under flow conditions [22]. Our goal is to achieve quantitative conversion of HTL to HCY and derivatization with OPA in a single run. Analysis of mixtures is accomplished by a simple (centrifugation-based) and elegant off-line step based on the different interactions of the analytes with fluorosurfactant (FSN)-modified gold nanoparticles (GNPs) [14]. To the best of our knowledge, this is the first automated flow assay for HTL reported in the literature.

## 2. Results and Discussion

### 2.1. Hydrolysis of HTL under Flow Conditions

**Figure 1:**
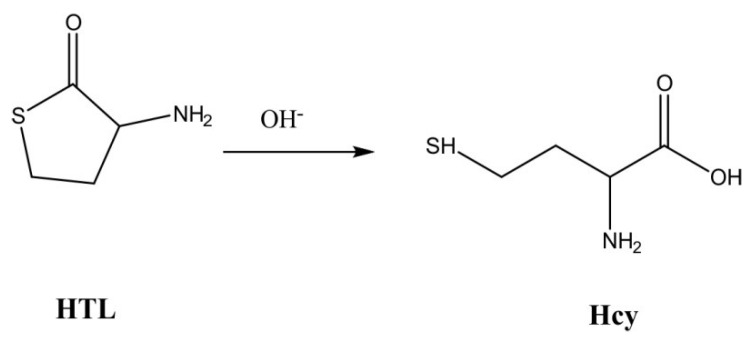
Hydrolysis of homocysteine thiolactone under alkaline conditions.

HCY reacts with OPA under flow conditions and in highly alkaline medium (0.5 mol L^−1^ NaOH [21]) to form a highly fluorescent derivative in the absence of nucleophilic reagents. The chemical system is specific in the presence of cysteine and other common amino acids and highly selective against histidine, histamine, and glutathione. On the other hand, HTL can react with the derivatizing reagent only after cleavage of the thiolactone ring to yield HCY (Figure 1) [22].

**Figure 2:**
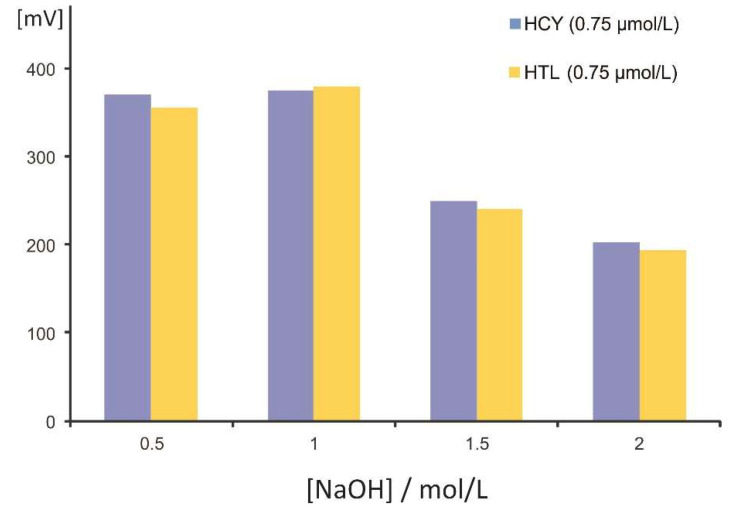
Effect of the concentration of NaOH on the hydrolysis of homocysteine thiolactone.

The potential of automating the hydrolysis and derivatization in a single step under zone fluidics was investigated using the setup described in Section 3.2. Equal amount concentrations of HCY and HTL (aqueous solutions of 0.75 μmol L^−1^ each) were processed sequentially using elevating concentrations of NaOH (0.5 to 2.0 mol L^−1^). The experimental results are depicted in Figure 2 and clearly demonstrate the effective hydrolysis of HTL (97–101%) at all NaOH levels (at [NaOH] > 1 mol L^−1^, the sensitivity decreased equally for both analytes). It is also worth mentioning that no heating of the reaction coil nor stopped-flow was necessary to improve the cleavage of the thiolactone ring, simplifying the on-line assay. Based on the findings in Figure 2, a concentration of NaOH of 1.0 mol L^−1^ was selected for further experiments.

In a following series of experiments, the effective cleavage of the thiolactone ring under flow conditions was investigated at the entire practical linearity range in the artificial urine matrix (2 to 30 μmol L^−1^). The experimental procedure involved the steps described in Section 3.4 under the “Analysis of HCY+HTL”. The ratio of the slopes of the curves (29.3 (±0.8) for HTL and 29.8 (±0.6) for HCY) indicated 98.3% conversion within the whole concentration range.

### 2.2. Separation of HCY and HTL

Since HCY and HTL react in a rather identical way with OPA/NaOH under flow conditions, simultaneous analysis can be carried out only after a simple and yet effective separation step.

GNPs have been evolved as viable alternatives for both sample preparation and sensor development in bioanalysis [23,24,25]. Fluorosurfactant-capped GNPs (FSN-GNPs) have proven to offer enhanced specificity and, most importantly, stability under high salinity conditions [26]. FSN interacts with the nanoparticles through the hydrophilic end of the molecule, while the hydrophobic chains remain dispersed in the solution [27,28]. Small molecules, such as HCY, can penetrate the FSN layer and interact with the GNPs, causing aggregation. Larger molecules are repelled through strong hydrophobic interactions, offering unique selectivity properties.

**Figure 3:**
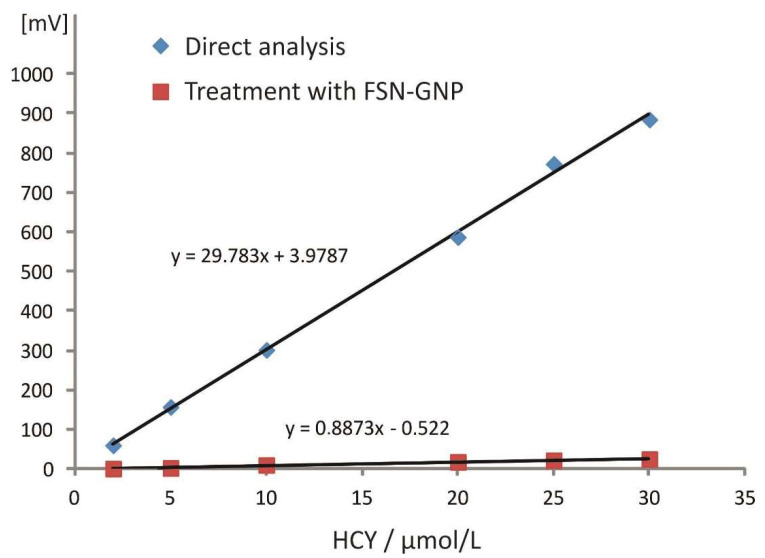
Study of the efficiency of the removal of homocysteine by the fluorosurfactant-capped gold nanoparticles.

On this basis, Huang and Cheng have reported quantitative removal of HCY (ca. 98%) using the FSN-GNP-based procedure described in Section 3.4 [14]. To verify their findings, artificial urine matrix spiked with HCY in the range of 2–30 μmol L^−1^ (final concentrations of 0.1–1.5 μmol L^−1^) were processed either directly or following the separation step. The experimental results are depicted in Figure 3. Based on the ratios of the slopes, ca. 97.1% removal of HCY was achieved.

**Figure 4:**
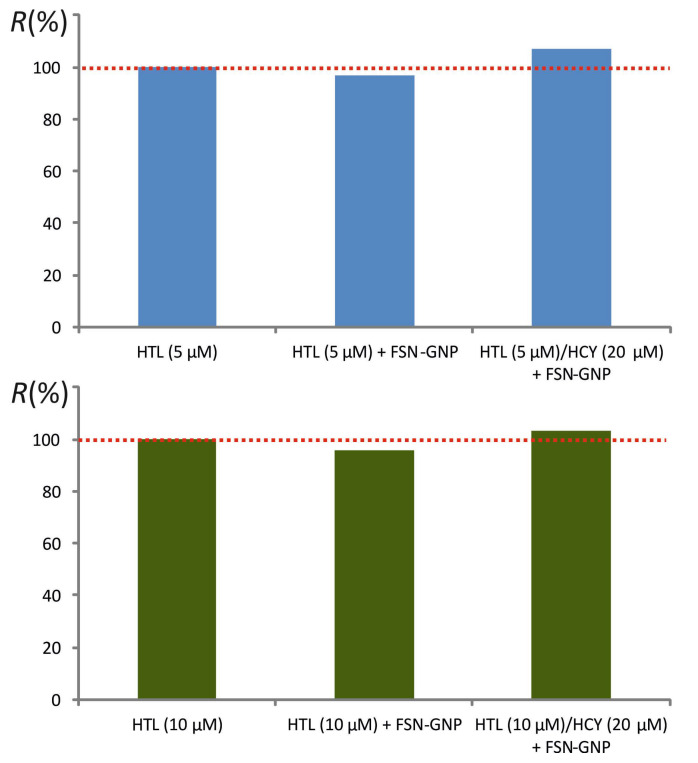
Recoveries of homocysteine thiolactone following treatment with fluorosurfactant-capped gold nanoparticles.

A second series of experiments confirmed the absence of interaction of the FSN-GNPs with HTL at two concentration levels, namely 5 and 10 μmol L^−1^. Repetitive separation experiments resulted in satisfactory recoveries in the range of 95–108%, both in the absence and in the presence of HCY (20 μmol L^−1^) (Figure 4).

The repeatability of the separation protocol was evaluated by processing mixtures of HTL (10 μmol L^−1^) and HCY (20 μmol L^−1^) through six replicate experiments (EXP1–EXP6), followed by analysis by the developed ZF method. The RSD was quite satisfactory, being <5% (Figure S1).

### 2.3. Analytical Figures of Merit

Linearity for both analytes was evaluated in artificial urine matrix following the experimental procedure described in Section 3.4. The respective regression equations in the range of 2–30 μmol L^−1^ (corresponding to 0.1–1.5 μmol L^−1^ in aqueous solutions) were obtained in a cumulative way by incorporating data from independent analyses through six nonconsecutive working days (36 data points for each analyte):

F(HCY) = 30.78 (±0.91) × [HCY] + 1.98 (±6.65), r = 0.991 F(HTL) = 29.12 (±0.63) × [HTL] + 1.24 (±5.52), r = 0.998(1)

The LOD for both analytes was estimated to be 0.6 μmol L^−1^ (based on the standard deviation of the intercept rule) and the LLOQ = 2 μmol L^−1^ (lower level of the calibration graph with residuals of <±10%). Both values refer to the artificial urine matrix considering the 20-fold dilution.

The within-day precision was evaluated by repetitive injections (*n* = 8) of HCY and HTL in artificial urine matrix at a low (2 μmol L^−1^) and a medium level (10 μmol L^−1^). The RSD values were in the range of 1.1 and 2.4% in all cases. The between-days precision was validated by obtaining independent calibration curves for both analytes within six nonconsecutive working days. The respective RSD values of the slopes were 5.2% (HCY) and 5.9% (HTL).

### 2.4. Analysis of HCY and HTL in Artificial Urine

The feasibility of the proposed procedure was evaluated by analysis of various mixtures of HCY and HTL in artificial urine matrix. Based on our previous findings, 20-fold dilution of the matrix is adequate to avoid matrix effects (see Section 3.4) [21]. The experimental results are included in Table 1 (samples S1–S10). As indicated by the percent recoveries, the incorporation of the hydrolysis of HTL in alkaline medium and the derivatization reaction with OPA in a ZF configuration combined with an effective separation pretreatment step offers satisfactory accuracy (R = 89–116%).

Potential applicability has also been examined in pooled human urine, focusing on the evaluation of the matrix effect. Real urine samples (see Section 3.4) were spiked with the analytes and processed at two dilution factors, namely 1:10 and 1:20. The experimental results were quite similar to our findings for artificial urine [21]. Based on the ratios of the slopes, the matrix effect at 1:10 dilution was calculated to be −22.6%, whereas 1:20 dilution offered an acceptable matrix effect of −5.1%. As can be seen in Table 1 (S11–S15), the percent recoveries ranged between 87 and 119%.

## 3. Materials and Methods

### 3.1. Instrumentation

The zone fluidics (ZF) instrumentation consisted of the following parts: a Minipuls3 peristaltic pump (Gilson, Middleton, WI, USA), a micro-electrically actuated 10-port valve (Valco, Brockville, ON, Canada), and an RF-551 flow-through spectrofluorimetric detector operated at high sensitivity (Shimadzu, Kyoto, Japan). The flow connections were made of PTFE tubing (0.5 or 0.7 mm i.d.), except for the connection used in the peristaltic pump that was made of Tygon tubing. The reaction coil (RC, 100 cm × 0.5 mm i.d.) was tightly wrapped around a metallic rod (10 cm × 4.6 mm i.d.) and was thermostated at the desired temperature (±0.1 °C) using an HPLC column heater (Jones Chromatography).

The ZF system was operated through the LabVIEW^®^, a home-developed program (National Instruments, Austin, TX, USA). Data acquisition (as peak heights) was carried out through the Clarity^®^ software (version 4.0.3, DataApex, Prague, Czech Republic).

### 3.2. Reagents and Materials

Homocysteine (HCY, Merck), homocysteine thiolactone (HTL, Sigma), *o*-phthalaldehyde (OPA, Fluka), NaOH (Sigma), and HCl (Sigma) were all of analytical grade. Doubly deionized water was produced by a B30 water purification system (Adrona SIA, Riga, Latvia).

The standard stock solutions of the analytes were prepared at a concentration level of 10 mmol L^−1^ by dissolving accurately weighted amounts in 50 mmol L^−1^ HCl and were kept at 4 °C. Working aqueous standards were prepared daily by serial dilutions in doubly deionized water in the range of 0.1–1.5 μmol L^−1^ for HCY and HTL (2–30 μmol L^−1^ in artificial urine). The OPA solution (*c* = 10 mmol L^−1^) was prepared by dissolving the appropriate amount in 500 μL of MeOH + 9500 μL water and was stable in light-protected vials for 5 working days when stored at 4 °C. The NaOH solutions were prepared at the required concentration levels in water (0.5–2 mol L^−1^).

FSN-modified GNPs were prepared following the procedure described by Huang and Cheng [14]. In brief, 54 μL of HAuCl_4_ (10% *m*/*v*, Sigma) was added rapidly to an aqueous solution of sodium citrate (60 mL, 0.075% *m*/*v*) under vigorous boiling and continuous stirring. The resulting mixture was heated under reflux for an additional 15 min to obtain a deep-red-colored solution (λ_max_ = 520 nm). Modification of the GNPs with FSN was carried out by adding 240 μL of the surfactant (10% *v*/*v*, Sigma) in the GNPs (60 mL) at room temperature and under stirring, following by storage at 4 °C.

The composition of the artificial urine matrix can be seen in Table S1. All compounds were mixed in deionized water and the pH of the solution was adjusted to 6.0 by addition of 1.0 mol L^−1^ hydrochloric acid.

### 3.3. ZF Procedure

**Figure 5:**
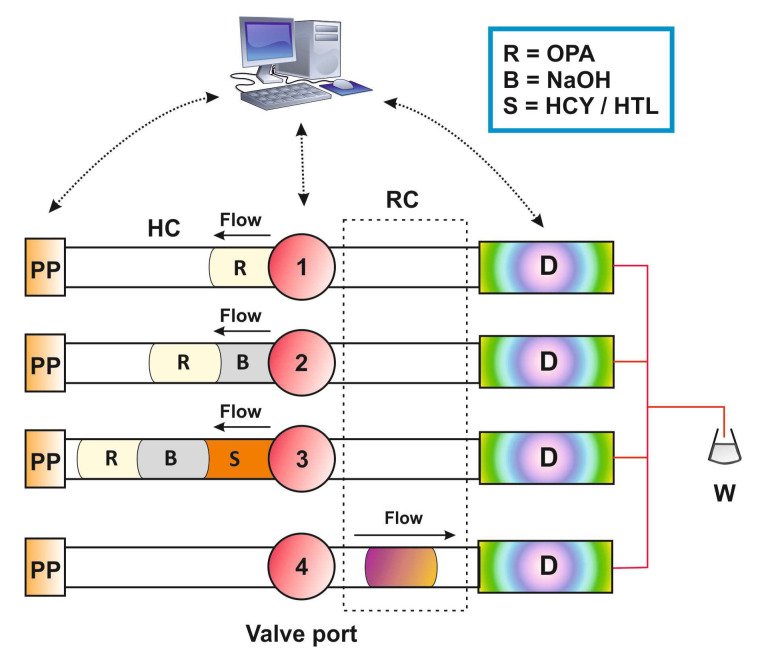
Schematic diagram of the zone fluidics setup: PP = peristaltic pump; HC = holding coil; RC = reaction coil; D = fluorimetric detector; W = waste.

The ZF procedure for the determination of HCY and HTL consisted of the following steps (Figure 5) [21]: 50 μL of OPA (10 mmol L^−1^), 50 μL of NaOH (1 mol L^−1^), and 150 μL of standards were aspirated in this order in the holding coil through the respective ports of the multi-position valve. The flow was reversed and the zones passed through a 100-cm-long reaction coil at a flow rate of 0.6 mL min^−1^, in which the HCY–OPA reaction product was formed (or hydrolysis and derivatization in the case of HTL). The derivatives were monitored fluorimetrically as peak heights at λ_ext_/λ_em_ = 370/480 nm.

HCY and HTL were determined in two successive runs; (i) in a first run, total HCY + HTL was determined without prior separation of the analytes, and (ii) HTL was quantified following separation using the FSN-GNP-based procedure in a second run. HCY was estimated by difference.

### 3.4. Preparation of Samples

Artificial urine matrix was prepared as described in Section 2.2 and aliquots were spiked with HCY, HTL, and their mixtures in the range of 2 to 30 μmol L^−1^. In an analogous way, a pooled human urine sample from apparently healthy volunteers (*n* = 6, members of the lab) was also utilized.

Analysis of HCY+HTL: 50 μL of the spiked matrix (artificial or human urine) was diluted to 1000 μL with water and analyzed under the ZF conditions proposed above.

Analysis of HTL: 50 μL of the spiked matrix (artificial or human urine) was diluted to 500 μL with water, followed by the addition of 500 μL of FSN-GNP solution. The mixture was allowed to react for 20 min and the aggregated nanoparticles were separated by centrifugation (18,000 rpm, 20 min). The supernatant was analyzed directly by the ZF procedure.

## 4. Conclusions

The present report offers—to our opinion—some interesting features: (i) this is, to the best of our knowledge, the first method for the assay of HTL using automated flow methods; (ii) hydrolysis in alkaline medium and derivatization of HTL with OPA are carried out rapidly and quantitatively in a single step; (iii) due to the rapid and on-line character of the flow scheme, potential side reactions that are favored under alkaline batch conditions (e.g., formation of 2,5-diketopiperazine [29]) are avoided; (iv) there is no need for elevated temperatures and time-consuming stopped-flow mode; (v) high specificity is achieved by reacting with OPA in the absence of nucleophilic compounds; (vi) HTL and HCY can be quantified in their mixtures following a simple and yet efficient separation step based on the selectivity of FSN-capped GNPs; (vii) sub-micromolar levels can be analyzed in aqueous solutions and low micromolar levels in diluted artificial and human urine without matrix interferences; (viii) application to real human urine requires either the use of a matrix-matched calibration curve or at least 20-fold dilution. Further investigation is required in order to develop an analyte preconcentration scheme that will enable the quantification of the analytes in human urine at the nanomolar level.

## Figures and Tables

**Table 1:** Determination of HCY/HTL in artificial urine (S1–S10) and in human urine (S11–S15).

Sample	HTL (μmol L^−1^)	Recovery (%)	HCY (μmol L^−1^)	Recovery (%)
S1	—	—	2	89 (±3)
S2	2	112 (±5)	—	—
S3	5	95 (±3)	5	109 (±5)
S4	5	109 (±5)	10	97 (±3)
S5	5	108 (±4)	20	110 (±4)
S6	—	—	10	91 (±4)
S7	10	115 (±5)	—	—
S8	10	112 (±4)	10	102 (±3)
S9	20	92 (±5)	5	116 (±5)
S10	20	90 (±2)	—	—
S11	5	87 (±5)	—	—
S12	5	89 (±4)	5	85 (±5)
S13	—	—	10	114 (±6)
S14	10	119 (±3)	5	107 (±4)
S15	10	102 (±4)	10	90 (±6)

## Data Availability

Not applicable.

## Supplementary Materials

The following supporting information can be downloaded at: https://www.mdpi.com/article/10.3390/molecules27072040/s1, Figure S1: Repeatability of the removal of Homocysteine in the presence of Homocysteine thiolactone (six independent experiments, EXP1-EXP6); Table S1: Composition of the artificial urine matrix.

## Sample Availability

Samples of the compounds are not available from the authors.
