# Supplementary material for: Single-Step Hydrolysis and Derivatization of Homocysteine Thiolactone Using Zone Fluidics: Simultaneous Analysis of Mixtures with Homocysteine Following Separation by Fluorosurfactant-Modified Gold Nanoparticles
Source: Molecules. 2022 Mar 22;27(7):2040. doi: 10.3390/molecules27072040 (PMC9000217; doi:10.3390/molecules27072040)
Supplement: Supplementary file 1 [file molecules-27-02040-s001.zip › molecules-1614550-supplementary.pdf]

Supplementary Material

Single-Step Hydrolysis and Derivatization of Homocysteine Thiolactone Using Zone Fluidics: Simultaneous Analysis of Mixtures with Homocysteine Following Separation by Fluorosurfactant-Modified Gold Nanoparticles

Apostolia Tsiasioti [1], Constantinos K. Zacharis [2] and Paraskevas D. Tzanavaras [1,*]

[1] Laboratory of Analytical Chemistry, School of Chemistry, Faculty of Sciences, Aristotle University of
Thessaloniki, 54124 Thessaloniki, Greece; atsiasioti@gmail.com
[2] Laboratory of Pharmaceutical Analysis, Department of Pharmaceutical Technology, School of Pharmacy, Aristotle University of Thessaloniki, 54124 Thessaloniki, Greece; czacharis@pharm.auth.gr
* Correspondence: ptzanava@chem.auth.gr; Tel.: +30-23-1099-7721

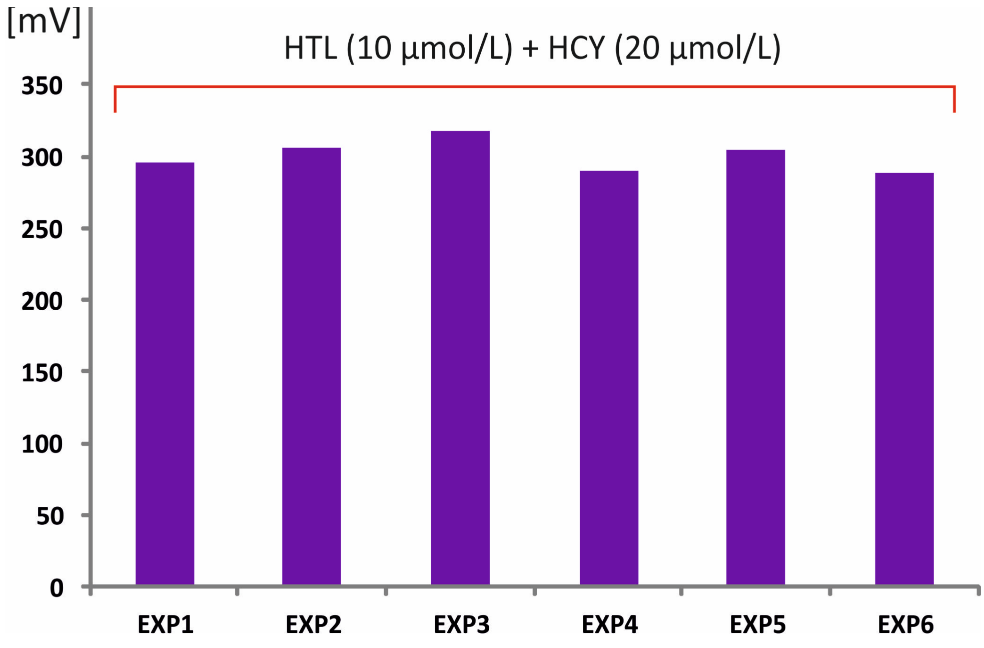

Figure S1. Repeatability of the removal of Homocysteine in the presence of Homocysteine thiolactone (six independent experiments, EXP1-EXP6).

Table S1. Composition of the artificial urine matrix.

**Species**	**Amount concentration (mmol L^{-1})**
Lactic acid	1.1
Citric acid	2.0
Sodium bicarbonate	25
Urea	170
Calcium chloride	2.5
Sodium chloride	90
Magnesium sulfate	2.0
Sodium sulfate	10
Potassium hydrogen phosphate	7.0
di-Potassium hydrogen phosphate	7.0
Ammonium chloride	25
